# Letter to Editor: Mental health after orchiectomy: Systematic review and strategic management

**DOI:** 10.1080/20905998.2025.2494179

**Published:** 2025-04-21

**Authors:** Hery Sumasto, Sari Luthfiyah, Triwiyanto Triwiyanto, Nurwening Tyas Wisnu

**Affiliations:** a Department of Midwifery, Poltekkes Kemenkes Surabaya, Surabaya, Indonesia; b Department of Nursing, Poltekkes Kemenkes Surabaya, Surabaya, Indonesia; c Department of Electromedical Technology, Poltekkes Kemenkes Surabaya, Surabaya, Indonesia

## Abstract

This letter to the editor addresses the article ‘Mental Health After Orchiectomy: Systematic Review and Strategic Management’ by Shokaier et al, which reviews the psychological impacts of orchiectomy, including depression, anxiety, and reduced quality of life. The letter acknowledges the importance of the review but highlights gaps in the study, such as the lack of exploration into how cultural and socioeconomic factors influence mental health outcomes. It also points out the variability introduced by different psychological assessment tools used in the studies, which may limit the robustness of the conclusions. The letter suggests integrating artificial intelligence (AI) to analyze large datasets on post-orchiectomy mental health, proposing that AI-driven models could identify risk factors more precisely and offer personalized interventions. Additionally, the letter calls for expanding research to include a longitudinal perspective to understand the long-term psychological effects of orchiectomy. The author commends the original work while urging further studies to address these gaps, thereby advancing the understanding and management of post-orchiectomy mental health.

## Dear editors

I am writing in response to the article titled ‘Mental Health After Orchiectomy: Systematic Review and Strategic Management’ by Shokaier et al. [1]. I found this article to be a significant contribution to the field, as it highlights the psychological consequences of orchiectomy, a procedure often overlooked in the context of mental health. The authors’ systematic review effectively synthesizes various studies and emphasizes the need for a multifaceted approach to manage mental health challenges, such as depression, anxiety, and diminished quality of life, which patients often face after undergoing this procedure.

However, I believe there are a few limitations in the study that should be addressed. Firstly, the article mentions the psychological impacts of orchiectomy but lacks a detailed exploration of how these impacts vary across different cultural and socioeconomic backgrounds. While the review acknowledges that psychological outcomes may differ, it does not provide substantial data on how these factors influence mental health outcomes, which would be critical for a more inclusive understanding of the procedure’s impact. Secondly, the review heavily relies on studies that utilize different psychological assessment tools, which may introduce variability in the findings. The lack of standardized measures could compromise the ability to draw robust conclusions about the overall psychological impact of orchiectomy. Finally, while the article suggests potential therapeutic interventions, it does not fully explore the challenges patients face in accessing mental health support, particularly in lower-resource settings, which could further exacerbate mental health difficulties post-surgery.

To enhance the existing body of research, I would like to offer a few suggestions for future investigations. First, I propose integrating artificial intelligence (AI) tools to analyze large datasets on mental health outcomes post-orchiectomy [2]. AI-driven models can uncover deeper insights by identifying patterns in patient data, such as demographic variables, pre-existing mental health conditions, and post-surgical experiences [3]. This could provide a more precise understanding of who is at greatest risk for psychological distress. Additionally, AI could aid in the development of personalized mental health interventions based on individual patient profiles, improving both the accessibility and effectiveness of post-operative care [4]. Furthermore, I suggest expanding the study’s scope to include a longitudinal perspective, where researchers track mental health outcomes over several years. This could provide a clearer picture of the long-term psychological effects and help inform strategies for early intervention.

I commend the authors for their thorough review and their efforts to address a critical aspect of healthcare. The article undoubtedly adds value to the growing body of research on post-orchiectomy mental health, and I look forward to seeing future studies that explore these important issues further.

## Declaration of generative AI and AI-assisted technologies in the writing process

During the preparation of this work, the author(s) utilized [QuillBot and SciSpace] to refine the language without altering the scientific substance of the manuscript.
